# Wound Retractor Laparoscopic Port System for Laparoscopic Ovariectomy in *Panthera leo*

**DOI:** 10.3390/ani12060700

**Published:** 2022-03-10

**Authors:** Luca Lacitignola, Pietro Laricchiuta, Annarita Imperante, Claudia Acquafredda, Marzia Stabile, Annalaura Scardia, Marta Guadalupi, Caterina Vicenti, Alberto Crovace, Francesco Staffieri

**Affiliations:** 1 Dipartimento dell’Emergenze e Trapianti di Organo, Sez. Cliniche Veterinarie e P.A., Università Degli Studi di Bari, 70100 Bari, Italy; francesco.staffieri@uniba.it; 2 Zoosafari di Fasano, 72015 Brindisi, Italy; laris@libero.it; 3 Dottorato di Ricerca in Trapianti di Tessuti ed Organi e Terapie Cellulari, Università Degli Studi di Bari, 70100 Bari, Italy; annarita.imperante@uniba.it (A.I.); claudia.acquafredda@uniba.it (C.A.); marzia.stabile@uniba.it (M.S.); annalaura.scardia@uniba.it (A.S.); marta.guadalupi@uniba.it (M.G.); caterina.vicenti@uniba.it (C.V.); 4 Dipartimento di Scienze Mediche di Base, Neuroscienze e Organi di Senso, Università Degli Studi di Bari, 70100 Bari, Italy; alberto.crovace@uniba.it

**Keywords:** laparoscopy, ovariectomy, lionesses, wound retractor device, *Panthera leo*

## Abstract

**Simple Summary:**

The procedures are carried out using three portals, with a retractor platform at the umbilical port and cannulas inserted 3–4 cm cranial and caudal to the device at the midline. We evaluated the procedure’s duration as well as the intraoperative and early postoperative complications. The extraction of dissected ovarian tissue and annexes went off without complications. It is feasible and without intraoperative difficulties to employ a laparoscopic platform during three-portal operations for laparoscopic ovariectomy in adult obese lionesses. Because of the retractor, the 25 mm mini-laparotomy posed no entry-related issues. It also made retrieving thick ovaries and swiftly re-establishing the pneumoperitoneum simpler.

**Abstract:**

The aim of this study was to assess the feasibility and intraoperative complications of performing ovariectomies in African lionesses (*Panthera leo*) using a wound retractor laparoscopic platform. Six lionesses (*n* = 6) were included. The surgical procedures were carried out through three portals, with a retractor platform positioned at the umbilical port and cannulas placed 3–4 cm from the cranial and caudal regions to the device at the level of the midline. An ovariectomy was performed with a vessel-sealing device. We evaluated the surgery time and the intraoperative and early postoperative complications. The mean weight was 172.83 kg. The total surgery time was 49.33 min. The installation step took a mean of 10.33 min to complete. The mean ovariectomy time was 20 min. Controlled bleeding was observed at the tip of the uterine horn in two cases due to excessive tissue thickness. The retrieval of dissected ovarian tissue and annexes was easily performed. No other complications were observed. The use of the laparoscopic platform during three-portal surgeries for laparoscopic ovariectomy in adult overweight lionesses is feasible and without intraoperative problems. The retractor meant that there were no entry-related issues due to the 25 mm mini-laparotomy. It also made it simpler to extract thick ovaries and promptly re-establish the pneumoperitoneum.

## 1. Introduction

Big felids cared for in many zoos and sanctuary facilities breed so effectively that contraceptive methods are used to keep the population at a healthy size. Reproduction can be avoided by separating the sexes or through permanent sterilization [1], in addition to reversible contraception and gonadotropin-releasing hormone (GnRH) agonists [2,3,4,5], progestins implants [6], and vaccines [7]. Although reversible contraception is desirable since it permits natural social groupings to be preserved while maintaining the population’s genetic health [2], surgical contraception has been utilized in animals with various pathologies and medical disorders and in animals that should not be bred [1]. Furthermore, for individuals who are genetically well represented or for whom reproduction might entail potential health risks, permanent sterilization may be considered. Gonadectomy has been performed safely in lions since 1950 [8]. However, every surgical operation in big felids entails some inherent complications, both during surgery and in the postoperative period. 

Since big felids have a very deep and wide abdominal cavity in comparison to other carnivores, making it difficult to visualize and ligate the mesovarium, even with a large ventral midline incision [9,10]. In an effort to reduce the risk of suture line dehiscence and self-induced trauma, shorten recovery times, and reduce postoperative care, laparoscopic gonadectomy associated or not with hysterectomy is a relatively modern method that has been previously described [9,11,12].

Laparoscopic ovariectomies (OVE) have been described successfully in many big felids, and they are preferred to laparoscopic ovariohysterectomies (OVH) because they require smaller surgical ports and avoid the use of additional portals to perform broad ligament dissection and cervix ligation. Furthermore, laparoscopic OVE is recommended, since the ovary removal prevents the hormonal trigger that can lead to pyometra, a major cause of morbidity in captive felids [13]. However, if uterine disease has been diagnosed, the laparoscopically assisted surgery is strongly advised to limit the danger of uterine rupture during organ manipulation and exteriorization [13,14].

Laparoscopic OVE has been successfully described in big felids when using three portals [11,15,16] or single-portal techniques [10,17,18]. Both of these techniques use three tools, which eliminates the need for transabdominal ovarian pedicle suspension to the abdominal wall, which is required when performing a two-cannula ovariectomy, as described for dogs and cats [19]. The use of a single portal, on the other hand, necessitates a distinct learning curve, a higher level of surgical abilities, and articulated tools because the tight trajectory of straight instruments causes shaft interference and the loss of triangulation [20].

The use of laparoscopic wound retractors was described for the treatment of pyometra [21,22] and OVE/OVH [23], in several laparoscopically assisted procedures in dogs [23,24,25], and in cryptorchidectomy in horses [26]. The main advantage of this new technique is that the surgeon can easily interrupt the pneumoperitoneum by removing the cap, performing extracorporeal procedures, or removing a large specimen from the abdomen, and then easily resume the laparoscopic procedure after re-inserting the cap and re-insufflating the abdomen to induce pneumoperitoneum once more.

We hypothesized that using a wound retractor laparoscopic platform for laparoscopic ovariectomy in African lionesses (*Panthera leo*) would be possible, allowing us to complete the surgery in a reasonable length of time and with a low risk of intraoperative complication.

## 2. Materials and Methods

### 2.1. Animal Welfare

The study was authorized with written informed consent by the zoo’s owner (Leo 3000 S.p.a, c/o Zoosafari di Fasano, Brindisi, Italy). The laparoscopic procedures were performed by the first author (L.L.) and a zoo veterinary team. The study was approved by the Ethical Committee for Clinical studies of the Dipartimento dell’Emergenze e Trapianti di Organi of Università degli Studi di Bari, “Aldo Moro” (Approval no. 03/2022). The study was performed in January 2022.

### 2.2. Inclusion Criteria

Adult (>4 years old) lionesses (female *Panthera leo*) were determined to be suitable for laparoscopic OVE; subadults were excluded. Abdominal ultrasonography was conducted prior to surgery to assess the reproductive system and the existence of pregnancy. Animals in pregnancy or with uterine disorders were excluded from the sterilization program.

### 2.3. Capture and Anesthesia 

The day before the planned surgery, suitable animals were separated from the rest of the group in a different environment with the assistance of zoo personnel. A unique microchip number was used to identify each animal. Before surgery, the animals were fasted for 8 h and denied water for 3 h. The animals were darted with a combination of detomidine 0.05 mg/kg and ketamine 2 mg/kg. After complete immobilization was obtained, the lionesses were transported to the operative theatre. A 14-gauge intravenous cannula was inserted into the cephalic vein, and propofol (1–2 mg/kg) was given to permit the insertion of an 18 mm ID orotracheal tube. Heart rate, non-invasive arterial blood pressure, oxygen saturation (SpO2), capnography, and body temperature were all monitored under anesthesia. The animals were maintained with isoflurane in pure oxygen during the surgery with spontaneous ventilation. All animals received a single dosage of 10 mg methadone intramuscularly. In the event of a rapid change in HR, respiration rate, or blood pressure, an intravenous dosage of fentanyl 2 g/kg was administered as rescue analgesia. Cefazoline (20 mg/kg) and meloxicam (0.2 mg/kg) were given intravenously 30 min before the procedure. At the end of the procedure, isoflurane was discontinued, and the animals were moved to the recovery area with proper assistance. For anesthetic reversal, atipamezole was administered i.m. at 5 fold the detomidine dose.

### 2.4. Instruments and Surgical Setting

**Figure 1:**
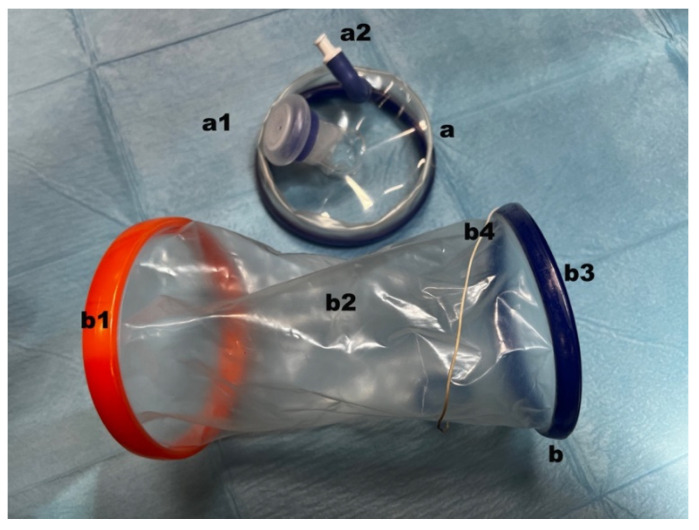
Images of wound retractor laparoscopic platform employed: (**a**) cap; (**a1**) instrumental port; (**a2**) CO_2_ inlet plug; (**b**) retractor; (**b1**) outer ring; (**b2**) polyurethan cylindrical sleeve; (**b3**) inner ring; (**b4**) removal tag.

The retractor (Endo Keeper model CG sized 265 mm length × 95 mm large Nelis, Bucheon Techno Park, Ssangyong, South Korea) and a cover were included in the ring wound retractor. The retractor component consisted of a doubled-over cylindrical sleeve made of transparent polyurethane that formed a conduit. Tension was established in the retraction sleeve by bringing the outer ring up to the inner one. This tension was necessary to retract the incision and make adequate space for the laparoscopic instrument. To remove the device from the incision following the surgery, a removal tag was put slightly above the inner ring. The cap was manufactured out of a separate outer ring with a specific gas intake and a flexible port for instrument insertion. The elastic port cap prevented gas loss and allowed the port to be opened during use (Figure 1).

### 2.5. Surgical Procedure

The lioness was positioned in dorsal recumbency on a custom made electrically tiltable table. The abdomen was clipped and aseptically prepared for surgery.

**Figure 2:**
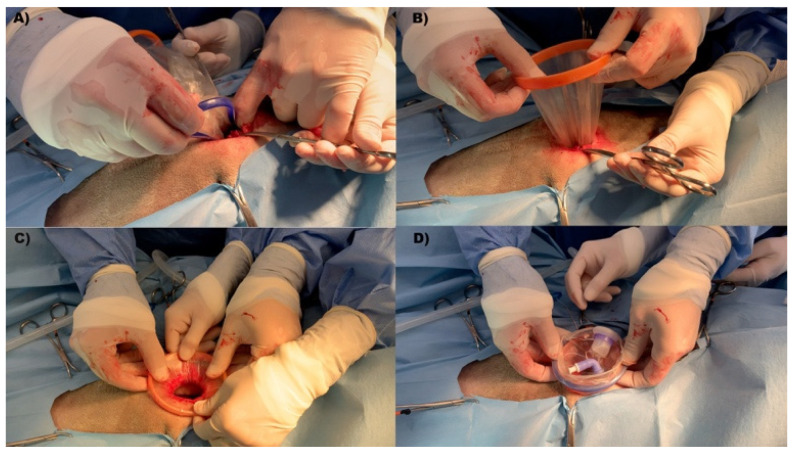
Installation of WRD laparoscopic platform. The WRD was inserted at midline ca. 1 cm caudal to the umbilical scar. Cranial is on the right. (**A**) Inner ring placement through 25 mm mini-laparotomy. The inner ring was squeezed to allow entrance in the abdominal cavity. (**B**) After the proper installation of the inner ring, the outer ring was adjusted for correct wound retraction and stable fixation to the abdominal wall. (**C**) Complete WRD installation and wound retraction. (**D**) The cap was mounted on the outer ring for tight portal sealing, CO_2_ gas tube connection, and instrument introduction.

The wound retractor port system was placed at 1 cm caudal to the umbilicus as the first port according to the following technique. A 25 mm-long skin incision was made to install the retractor, measured with a caliper. Blunt dissection was performed through the abdominal fascia. By using a set of Kelly’s forceps, the peritoneum was dissected, and the incision was enlarged using the surgeon’s finger. After mini-laparotomy, the inner ring was squeezed to facilitate entry into the abdominal cavity. The ring was advanced using the forceps, and the complete insertion of the inner ring was confirmed by digital palpation. The outer ring was rolled up until the right tension was achieved to secure the inner ring to the abdominal wall and create a retraction on the laparotomy (Figure 2). 

The cap was applied to the outer ring, and the valve was connected to inject CO_2_ gas to obtain an intra-abdominal pressure of 8–10 mm Hg.

**Figure 3:**
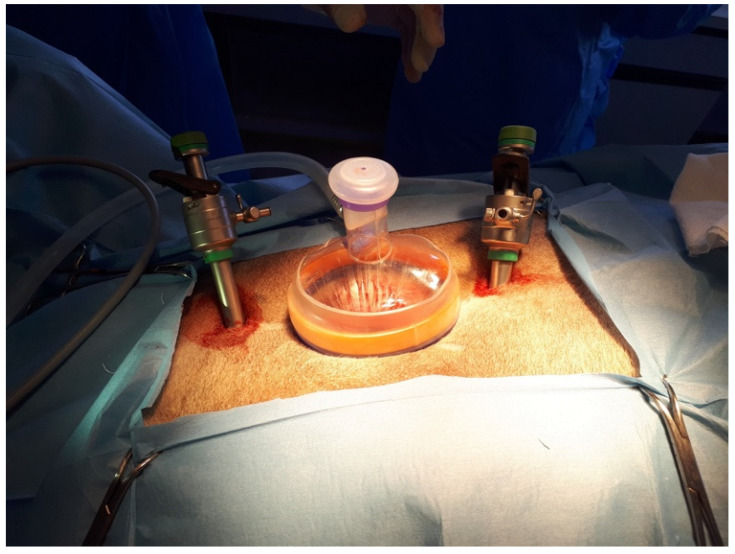
Complete installation of the three portals. Cranial is on the right. The cranial and caudal 12 mm cannulas were placed in the midline 3–4 cm from WRD border.

The laparoscope (10 mm diameter; 30° angle of vision, HOPKINS II, Karl Storz Endoskope GMBH & Co. KG, Tuttlingen, Germany) was placed in the abdominal cavity. The other two portals were created using two laparoscopic cannulas with a blunt trocar (11 mm diameter; 20 cm length; Karl Storz Endoskope), positioned in the midline, 3–4 cm cranial and caudal to the ring (Figure 3), under laparoscopic visualization.

**Figure 4:**
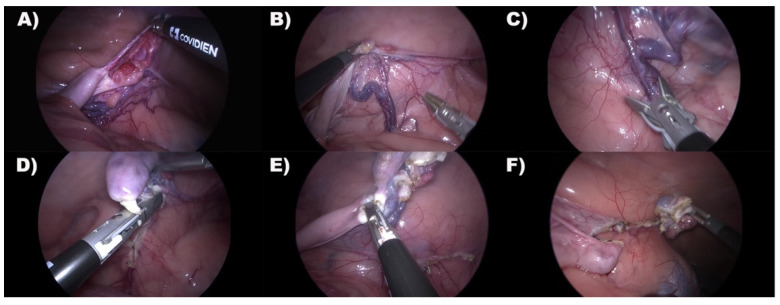
Representative laparoscopic images of right ovariectomy. Caudal is on the left. (**A**) Ovary inspection. (**B**) The ovary was grasped and suspended for ovarian vessel bound visualization. (**C**) The ovarian vessel bound was clamped with the vessel-sealing device axially for complete dissection. (**D**) The mesovarium was dissected with the vessel-sealing device. (**E**) The uterine horn was clamped and coagulated at the level of proper ovarian ligament with vessel-sealing device. Many coagulation cycles were applied to complete dissection. (**F**) Complete Ovary dissection.

Any complications related to the abdominal organs were recorded. The table was tilted by 45°, with the lionesses in left lateral recumbency, and the grasping forceps were introduced into the cranial cannula and the laparoscope into the central port one. The ovary was grasped and suspended, and the ovarian pedicle, proper ligament, and suspensory ligament were coagulated and transected using a 10 mm laparoscopic vessel-sealing device (LigaSure Atlas, Medtronic, Milan, Italy) placed in the caudal port. After complete dissection, the telescope was moved in the caudal port, and a set of 10 mm grasping forceps was introduced to grasp the ovary. The cap was removed from the platform and the ovary was retrieved through the wound retractor port. After re-establishing the pneumoperitoneum, and after the lionesses were repositioned on the opposite recumbency, the right ovariectomy was performed the same as the left one (Figure 4).

**Figure 5:**
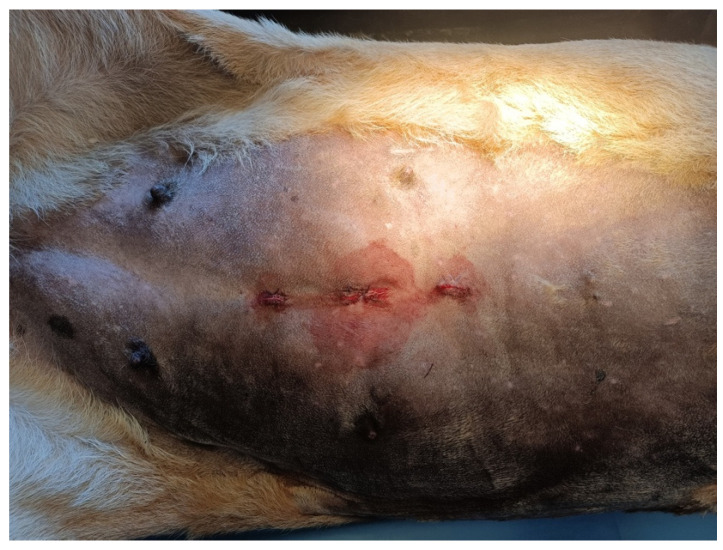
Portals sutured. Cranial is on the right.

After the second ovary was retrieved, the lioness was repositioned in dorsal recumbency, the retractor was removed by unrolling the external ring, and the internal ring was extracted by pulling the removal tag. The umbilical portal was closed with two layers of PDS (0-USP, Ethicon, Milan, Italy) with interrupted mattress suture. The 11 mm portals were sutured in one layer of PDS (0-USP, Ethicon, Milan, Italy) with a single interrupted mattress suture (Figure 5).

### 2.6. Clinical and Surgical Variables

The weight (kg, standard deviation (SD), range) was measured. Visual inspection was used to establish the body condition score, which was based on a previously defined scale [1].

The evaluated surgical variables were the total surgery time (min, mean ± SD, range) from the first skin incision to the last suture placement; the portal installation time (min, mean, ± SD, range) from the first skin incision until the final cannula insertion; the ovariectomy time (min, mean ± SD, range) from the last trocar placement to the second resected ovary retrieval. Specimens retrieved were measured with a caliper (mm, mean ± SD, range).

Furthermore, the number of attempts until correct cannula insertion, unintentional abdominal organ damage, and any intraoperative complications were all documented. The main surgeon awarded a semiquantitative score to the bleeding based on the following criteria: 0 indicates no bleeding; 1 indicates light bleeding (a few drops, self-limiting, with no effect on visibility); 2 indicates moderate bleeding (bleeding with minimal effect on visibility, necessitating additional application of the vascular sealing device); and 3 indicates serious bleeding (with a need for conversion to open surgery and hemostasis).

### 2.7. Postoperative Regimen and Aftercare

After being extubated, the animals were placed in a cage in a comfortable environment, separated from other animals, to recover from anesthesia. Once fully recovered, the lionesses were observed for variation of behavior and feeding, as well as indications of pain and suffering and wound dehiscence and bleeding. The animals were returned to the group and their normal habitat 24 h following their recovery.

## 3. Results

### 3.1. Clinical Study Population 

No pregnant animals were detected during pre-surgical evaluation. Six (*n* = 6) lionesses were included in the sterilization program. The mean weight was 172.83 kg (range 150–200 kg; SD 17.83). The median BCS was estimated to be 8 (range 7–9; SD 0.75). Table 1 summarize the results.

### 3.2. Surgery

Laparoscopic OVE was completed in all lionesses without conversion to open surgery. The rescue analgesia was not administered in any animal. The installation phase was completed in a mean time of 10.33 min (range 5–18 min; SD 4.59 min). The WRD was simply inserted through the 25 mm mini-laparotomy port. Nonetheless, the considerable amount of subcutaneous fat necessitated meticulous dissection in order to reach the linea alba. Even after the abdominal cavity was accessed, the excessive amount of falciform ligament fat required special attention for WRD inner ring insertion. However, when the ring was properly placed, the fat was pushed away, and optimum wound retraction was achieved in all cases. Although the subcutaneous amount of fat made the linea alba difficult to view, the insertion of the caudal cannula was not problematic. The cranial cannula placement, on the other hand, was more difficult due to the significant amount of fat in the falciform ligament, which required the cannula to penetrate through the ligament in certain cases for proper placement. No cases needed the re-insertion of cannulas, which were inserted on the first try, and no entry-related complications were observed. The cannula location allowed optimal triangulation, with good organ manipulation, without the interference of instruments. The employment of a motorized custom-made surgical table eased the tilting maneuvers necessary for ovary vision and manipulation at a 45° angle. 

The mean ovariectomy time was 20 min (range 16–30 min; SD 5.17 min). The coagulation of ovarian vessels was completed correctly with the aid of a vessel-sealing device, even when the vessels were significantly wide, and the dissection of proper ligament and mesovarium was conducted without difficulties. The dissection from the cranial uterine horn, on the other hand, necessitated many applications of the vessel-sealing device. We noticed tissue sliding from the instrument’s jaws and inadequate coagulation, necessitating additional coagulation cycles in some cases. In fact, we noticed grade 2 hemorrhage from the cranial uterine horn in two cases, which necessitated several coagulations and bleeding monitoring. Nonetheless, the bleeding was successfully managed and did not necessitate conversion. The ease with which the platform cap could be removed and reinserted during the ovary extraction maneuver allowed the pneumoperitoneum to be restored to the correct pressure without the need for platform repositioning. The ovary retrieval procedure was simple, with smooth passage from the retractor and no need to widen the portal. The mean size of specimens was 42.1 mm in length (range 24.8–61.9 mm; SD 14.4) and 30.7 mm in width (range 14.7–46.1 mm; SD 12.6). The total surgery time was 49.33 min (range 40–61 min; SD 8.61). Table 1 summarize the observed surgical variables.

### 3.3. Postoperative Evaluation

All animals recovered from anesthesia without issues and were returned to the rest of the group after 24 h.

Postoperatively, no animals showed signs of anorexia, reluctance to move, isolation, or altered social behavior. Unfortunately, physical clinical examinations were not performed for all operated animals in the mid- or long term postoperatively to prevent re-capture or physical and chemical restraint, which might cause further stress to the animals.

## 4. Discussion

The feasibility, surgical time, and acute perioperative complications of a three-portal approach to laparoscopic OVE in adult, overweight female lionesses (*Panthera leo*) were studied in this study. We used a wound retractor laparoscopic platform at the umbilical portal for the first time, rather than the 11 mm cannula [11,12,16,18] or single-port multi-cannulated platform, as previously reported for big felids [10,17,18].

All adult females with a mean weight of more than 170 kg and a BCS score of 8 were included in the study cohort. All animals were overweight, with substantial abdominal width and depth, according to the previously reported BCS scale for African lions [1]. Furthermore, the subcutaneous and falciform ligament fat in overweight lionesses were significant enough to necessitate some special attention, requiring the elongation of surgical time, to avoid entry-related complications and inadequate visualization, as previously described [10,16,18]. In this investigation, we used a wound retractor laparoscopic platform to control these issues. The WRD was simply placed via a 25 mm mini-laparotomy, which provided sufficient space for correct subcutaneous dissection to reach the linea alba for access to the peritoneal cavity. Furthermore, after the peritoneum had been incised, the falciform ligament was readily dissected or pushed aside, and the WRD could be firmly secured to the abdominal wall, resulting in a radial (360°) retraction on the mini-laparotomy. We also advocate using cannulas with a diameter of 11 mm and a length of 20 cm for the cranial and caudal instrumental ports. Long cannulas, in particular, reduced the possibility of unintentional cannula sliding back during surgical maneuvers or tilting the animals to 45° recumbency. Furthermore, the cranial portal can be passed via or beneath the falciform ligament while being careful not to inadvertently injure the spleen by using blunt trocars and under direct laparoscopic vision, as well as avoiding removing the cannula. Although the use of a single-port multi-cannulated laparoscopic port has previously been proposed in these species [10,16,18], the use of the platform described in other reports (SILS port) in an obese adult lioness may meet certain technical difficulties. The platform’s length is only available in specific sizes, but the WRD allows you to change the distance between the inner and outer rings and the patient’s abdominal wall by up to 265 mm. Furthermore, the use of a single-port access with a multi-cannulated platform restricts the size and number of instruments. Indeed, the number of cannulas available is restricted to one 12 mm and two 5 mm, necessitating the employment of a 5 mm telescope and a 10 mm vessel-sealing device, or a 10 mm telescope and a 5 mm vessel-sealing device. As a result, surgeons may opt to use a 5 mm telescope with possible poor illumination of the large abdominal cavity of adult patients. In the other hand, using 5 mm vessel-sealing devices could create insufficient force when clamping thick tissues, especially on the tip of the uterine horn. We discovered that in lionesses with a large tip of the uterine horn, even the 10 mm vessel-sealing device required many application and coagulation cycles, resulting in insufficient hemostasis and the requirement for further coagulation cycles and bleeding monitoring in two cases. We also noticed that the thick tissues of the uterine horn slipped from the instruments’ jaws in some circumstances. We reasoned that using tools with longer jaws (more than 22 mm) would result in improved coagulation and compression. However, the use of various vessel-sealing devices besides LigaSure Atlas needs to be considered further in future studies. The portal position chosen in our investigation allowed us to use conventional shaft length (37 cm) equipment, eliminating the usage of longer instruments (43 cm). To reduce the likelihood of difficulties reaching the ovary, we recommend that instrumental ports be no more than 8–9 cm from the umbilical scar. Although portal reduction and portal size were suggested in veterinary laparoscopy [19], the use of the multiport approach in our study allowed for good tissue manipulation owing to optimal instrument triangulation and avoided suspending the ovary to the abdominal wall. We did not contemplate the two-portal procedure due to the large thickness of the abdominal wall, which prevented the needle from passing through for the placement of suspension sutures. Furthermore, in large felids, the utilization of single-portal approaches has been noted to have certain technical challenges. In particular, other authors experienced instrument collision and the loss of triangulation in tigers and lionesses, resulting in the surgical time being lengthened [10,17,18]. In our study, the mean total surgical time was 49 min. This result was consistent with prior research in which big and obese animals were operated upon [10,18]. The most time-consuming phase during the operation was the ovariectomy. We suppose that the main cause was the employed vessel-sealing device, which required several applications to complete the ovary dissections. The mean ovarian size was 42 × 30 mm. Although the ovaries were removed from the abdomen during the ovariectomy time, this procedure had little impact on this phase. In fact, even with large specimens, the application of WRD enabled the extraction of harvested ovaries via the mini-laparotomy without difficulty. In fact, the radial retraction of the surgical site allowed the specimens to pass through with minimal pressure and reduced tissue trauma. The WRD implanted through the 25 mm mini-laparotomy eliminates the need for the incision to be enlarged. In addition, because it removes the need for disposable retrieval bags (USD 35–75), the wound retractor provides a low-cost (USD 20–30) alternative to standard port devices. Furthermore, while being designed to be a disposable device, the wound retractor with a cap can be reused several times after cold sterilization. The plastic layer that covers the incisions protects the incision tissues and prevents dehydration while also providing a smooth surface that facilitates extraction. After ovary extraction, the WRD must be closed again to reestablish a firm seal for the pneumoperitoneum required for the second ovary removal, as described for other single-port platforms [18]. However, the platform cap is simply applied and removed, making the operation easy and time-saving.

There were no complications during the early postoperative period. Unfortunately, due to the big felids’ aggressivity and risks during management, the direct examination of wounds was impossible, and in accordance with the zoo management’s policy and zoo vets’ directions, we excluded further chemical restriction in mid- and long-term follow-ups, limiting postoperative evaluation to observing from afar and the evaluation of variations in behavior and appetite, which represents an important limitation of this study. Another limitation was the lack of a control group to examine alternative portal configurations or comparison in the use of other vessel-sealing devices, and more research should be conducted.

## 5. Conclusions

According to our findings, using a WRD laparoscopic platform during three-portal procedures for laparoscopic ovariectomy in adult overweight lionesses is possible and free of intraoperative complications. However, the surgeon should consider using vessel-sealing devices with jaws able to adequately coagulate a thick uterine horn. Because of the 25 mm mini-laparotomy, the WRD did not lead to any entry-related problems. It also made it easier to retrieve thick ovaries and re-establish the pneumoperitoneum quickly and easily. As a result, the WRD laparoscopic platform might be an appropriate technology to use for laparoscopic ovariectomy in adult obese lionesses.

## Figures and Tables

**Table 1:** Weight, BCS, and surgical variables. The table summarize weight (kg), body condition score, total surgery time (min) from the first skin incision to the last scheme 45 recumbency. Time variables are expressed in min, mean, median, standard deviation (SD), and range.

	Mean	Median	SD	Minimum	Maximum
Weight	172.83	167.50	17.83	150.00	200.00
BCS	7.83	8.00	0.75	7.00	9.00
Installation time	10.33	9.00	4.59	5.00	18.00
Ovariectomy time	20.00	18.00	5.17	16.00	30.00
Total surgery time	49.33	47.50	8.61	40.00	61.00

## Data Availability

Not applicable.
